# Retrospective cohort study of nanoparticle albumin-bound paclitaxel plus ramucirumab versus paclitaxel plus ramucirumab as second-line treatment in patients with advanced gastric cancer

**DOI:** 10.1186/s12885-020-07614-6

**Published:** 2020-11-16

**Authors:** Mashiro Okunaka, Daisuke Kotani, Ken Demachi, Akihito Kawazoe, Takayuki Yoshino, Toshikatsu Kawasaki, Kohei Shitara

**Affiliations:** 1 Department of Pharmacy, National Cancer Center Hospital East, Kashiwa, Japan; 2 Department of Gastrointestinal Oncology, National Cancer Center Hospital East, 6-5-1 Kashiwanoha, Kashiwa, Chiba, 277-8577 Japan

## Abstract

**Background:**

Nanoparticle albumin-bound paclitaxel (nab-PTX) has shown non-inferiority to paclitaxel (PTX) as second-line therapy for advanced gastric cancer (AGC) with fewer infusion-related reactions. The efficacy and safety of nab-PTX plus ramucirumab (RAM) was reported in a phase II trial; however, there is no randomized trial comparing this regimen with PTX plus RAM in patients with AGC. This retrospective study aimed to investigate the efficacy and safety of nab-PTX plus RAM versus PTX plus RAM in patients with AGC.

**Methods:**

This study included patients with AGC who received nab-PTX plus RAM from September 2017 to January 2019 or PTX plus RAM from June 2015 to August 2017 as second-line chemotherapy in our hospital.

**Results:**

A total of 113 and 138 patients who received nab-PTX plus RAM and PTX plus RAM, respectively, were analyzed. Median progression-free survival (PFS) was 3.9 months (95% confidence interval [CI]: 3.4–4.3) in the nab-PTX plus RAM group and 3.9 months (95% CI: 3.1–4.7) in the PTX plus RAM group (hazard ratio [HR]: 1.08; 95% CI: 0.83–1.40; *P* = 0.573). Median overall survival (OS) was 10.9 months (95% CI: 9.3–12.7) in the nab-PTX plus RAM group and 10.3 months (95% CI: 8.5–12.0) in the PTX plus RAM group (hazard ratio: 0.82; 95% CI: 0.61–1.10; *P* = 0.188). In patients with moderate/massive ascites, favorable outcomes for progression-free survival were observed in the nab-PTX plus RAM group compared with the PTX plus RAM group. Although anemia and fatigue (any grade) were more frequent in the nab-PTX plus RAM group, discontinuation of study treatment was not increased in the nab-PTX plus RAM group. There was no occurrence of hypersensitivity reaction in the nab-PTX plus RAM group, while two patients (1.4%) experienced grade 3 hypersensitivity reactions in the PTX plus RAM group.

**Conclusions:**

The combination of nab-PTX plus RAM showed a similar efficacy and safety profile to PTX plus RAM as second-line treatment for patients with AGC.

**Supplementary Information:**

The online version contains supplementary material available at 10.1186/s12885-020-07614-6.

## Background

Gastric cancer remains the third-leading cause of cancer-related mortality worldwide [1, 2].

While the combination of platinum agents plus fluoropyrimidine has been established as first-line chemotherapy for unresectable AGC [2–5], RAM (an anti-vascular endothelial growth factor receptor 2 antibody) plus PTX demonstrated survival benefit versus PTX alone as second-line chemotherapy in the RAINBOW trial [6, 7].

Nab-PTX is a 130-nm nanoparticle formulation that links albumin to PTX, rendering it soluble. Owing to its improved water solubility, nab-PTX is free of polyethoxylated castor oil, which minimizes the risk of hypersensitivity reactions without premedication [8–11]. Furthermore, as this formulation does not require the use of hydrated alcohol as a solvent, it can be used in patients with alcohol intolerance. A recent randomized phase III trial (ABSOLUTE) showed that weekly nab-PTX was non-inferior to weekly PTX in terms of OS, with a lower incidence of hypersensitivity reactions [12]. This evidence led to the approval of weekly nab-PTX in Japan in August 2018. In addition, a phase II trial investigating the combination therapy of nab-PTX plus RAM showed promising activity and manageable toxicity in patients with previously treated AGC [13]. Based on these results, nab-PTX plus RAM is considered an option for second-line chemotherapy in patients with AGC [2, 3]. However, there is no randomized study comparing nab-PTX plus RAM versus PTX plus RAM. Therefore, the aim of this study was to investigate the efficacy and safety of nab-PTX plus RAM compared with PTX plus RAM as second-line chemotherapy in patients with AGC in clinical practice.

## Methods

### Study design and patients

We retrospectively reviewed the medical records of consecutive patients with AGC who received nab-PTX plus RAM or PTX plus RAM at the National Cancer Center Hospital East, Kashiwa, Japan. The study protocol of this retrospective analysis was approved by the institutional review board of the National Cancer Center Hospital East. Informed consent requirement was waived due to the study's observational retrospective design, with opt-out opportunity provided at the institution's website.

The eligibility criteria for patients were as follows: aged ≥18 years; diagnosed with histologically confirmed unresectable AGC; having history of previous treatment with fluoropyrimidine-containing first-line chemotherapy; received concurrent treatment with nab-PTX plus RAM from September 2017 to January 2019 or PTX plus RAM from June 2015 to August 2017.

The main exclusion criteria were as follows: other histology; histologically proven neuroendocrine carcinoma; treatment with nab-PTX plus RAM or PTX plus RAM as third or later line; and history of previous treatment with taxanes.

### Study procedures

The nab-PTX plus RAM regimen consisted of nab-PTX 100 mg/m^2^ intravenously over 30 min on days 1, 8, and 15 plus RAM 8 mg/kg intravenously on days 1 and 15 in a 28-day cycle. Premedication was only chlorpheniramine 5 mg before RAM infusion on days 1 and 15 (https://www.accessdata.fda.gov/drugsatfda_docs/label/2013/021660s037lbl, https://www.ema.europa.eu/en/medicines/human/EPAR/abraxane). The PTX plus RAM regimen consisted of PTX 80 mg/m^2^ intravenously over 30 min after premedication with dexamethasone 6.6 mg, chlorpheniramine 5 mg, and famotidine 20 mg on days 1, 8, and 15 plus RAM 8 mg/kg intravenously on days 1 and 15 in a 28-day cycle. Dose modification and interruption of treatment were decided by each investigator based on the criteria reported in clinical trials [6, 13].

The following baseline characteristics were collected for each patient: age, sex, Eastern Cooperative Oncology Group performance status, history of previous gastrectomy, history of adjuvant chemotherapy, time to progressive disease during first-line chemotherapy, human epidermal growth factor receptor 2 status, histology, metastatic sites, and amount of ascites.

### Outcomes

PFS was defined as time from the initiation of study treatment to disease progression or death from any cause. OS was defined as time from the initiation of study treatment to death due from any cause. Overall response rate (ORR) was defined as the proportion of patients who had a complete or partial response to the study treatment, and disease control rate (DCR) was defined as the proportion of patients who had a complete response, partial response or stable disease lasting > 6 weeks from the initiation of study treatment. Tumor response was assessed by each investigator in accordance with the Response Evaluation Criteria in Solid Tumors version 1.1. Adverse events were graded in accordance with the Common Terminology Criteria for Adverse Events version 4.03 [14]. The amount of ascites was defined using computed tomography as follows: none, small (limited to the pelvic cavity or around the liver), moderate (neither small nor massive), or massive (continuous ascites from the surface of the liver to the pelvic cavity). These definitions were used in previous studies [15].

### Statistical analysis

PFS and OS were compared between treatment groups using the log-rank test with a two-sided significance level of *P* = 0.05. HR and corresponding 95% CI were determined using a Cox proportional hazards model. Survival curves were generated using Kaplan–Meier estimates. ORR, DCR, and safety analyses between treatment groups were performed using Fisher’s exact test. Follow-up time was defined as time from the initiation of study treatment until the last follow-up date for censored cases. Statistical analyses were performed using the IBM SPSS statistics version 22.0 (IBM Corp, Armonk, NY, USA) software, and two-sided *P* < 0.05 denote statistically significant differences.

## Results

### Patients

**Fig. 1:**
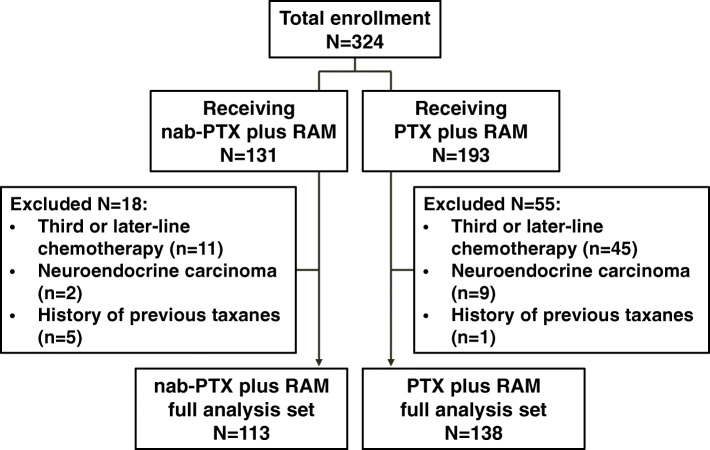
CONSORT diagram. Among 324 patients, 131 patients received nab-PTX plus RAM chemotherapy, and 193 patients received PTX plus RAM chemotherapy. Finally, 113 and 138 patients in the nab-PTX plus RAM and PTX plus RAM groups, respectively, were analyzed

A total of 131 and 193 patients received nab-PTX plus RAM and PTX plus RAM, respectively. Eighteen and 55 patients were excluded from the nab-PTX plus RAM and PTX plus RAM groups, respectively. Finally, 113 and 138 patients in the nab-PTX plus RAM and PTX plus RAM groups, respectively, were analyzed (Fig. 1). Baseline characteristics were generally well balanced between the two groups. Although the proportion of patients with ECOG PS 0 or HER2 negative were numerically higher, there were no significant differences (Table 1). Of the patients, 94.1 and 94.2% had received platinum agents in the nab-PTX plus RAM and PTX plus RAM groups, respectively. At the time of analysis in December 2019, the median follow-up was 16.8 and 37.9 months in the nab-PTX plus RAM and PTX plus RAM groups, respectively. Of the patients, 75.2 and 58.0% in the nab-PTX plus RAM and PTX plus RAM groups, respectively, received subsequent antitumor therapy, including anti-programmed cell death 1/programmed cell death 1 ligand 1 (anti-PD-1/PD-L1) inhibitor (57.5% vs. 26.8%, respectively), irinotecan (31.9% vs. 47.1%, respectively), platinum re-challenge (7.1% vs. 18.1%, respectively), and investigational agents in clinical trials (22.1% vs. 20.3%, respectively,) (Table S1).

**Table 1:** Patient characteristics

		nab-PTX plus RAM		PTX plus RAM		*P* value
		*N* = 113	%	*N* = 138	%	
Age	Median (range)	67 (25–84)		69 (40–85)		0.16
	> 65	71	62.8	96	69.6	0.261
Sex	Male	78	69	88	63.8	0.381
	Female	35	31	50	36.2	0.381
ECOG PS	0	85	75.2	88	63.8	0.051
	1	25	22.1	43	31.2	0.109
	> 2	3	2.7	7	5.1	0.261
Previous gastrectomy	Yes	35	31	41	29.7	0.828
	No	78	69	97	70.3	0.828
Recurrence during adjuvant chemotherapy		6	5.3	8	5.8	0.867
Time to progressive disease on first-line therapy	< 6 months	75	66.4	78	56.5	0.112
	> 6 months	38	33.6	60	43.5	0.112
HER2	Positive	13	11.5	23	16.7	0.246
	Negative	99	87.6	112	81.1	0.165
	Unknown	1	0.9	3	2.2	0.39
Histology	Diffuse	69	61.1	82	59.4	0.792
	Intestinal	43	38.1	52	37.7	0.952
	Mix, Missing	1	0.9	4	2.9	0.254
Number of metastatic sites	0–1	60	54	62	44.9	0.198
	2	33	28.3	48	36.2	0.347
	> 3	20	17.7	28	18.9	0.604
Metastatic site	Liver	32	28.3	46	33.3	0.393
	Lung	18	15.9	19	13.8	0.631
	Lymph node	54	47.8	74	53.6	0.358
	Peritoneum	70	61.9	80	58	0.523
	Others	13	11.5	26	18.8	0.11
Prior therapy	Fluoropyrimidine	113	100	138	100	–
	Platinum	107	94.7	130	94.2	0.867
	Trastuzumab	10	8.8	21	15.2	0.127
Ascites	None	59	52.2	75	54.3	0.736
	Small	22	19.5	20	14.5	0.293
	Moderate	14	12.4	15	10.9	0.708
	Massive	18	15.9	28	20.3	0.374
	None/small	81	71.7	95	68.8	0.625
	Moderate/massive	32	28.3	43	31.2	0.625

*nab-PTX* nanoparticle albumin-bound paclitaxel, *RAM* ramucirumab, *HER2* human epidermal growth factor receptor 2, *ECOG PS* Eastern Cooperative Oncology Group performance status

### Efficacy

**Fig. 2:**
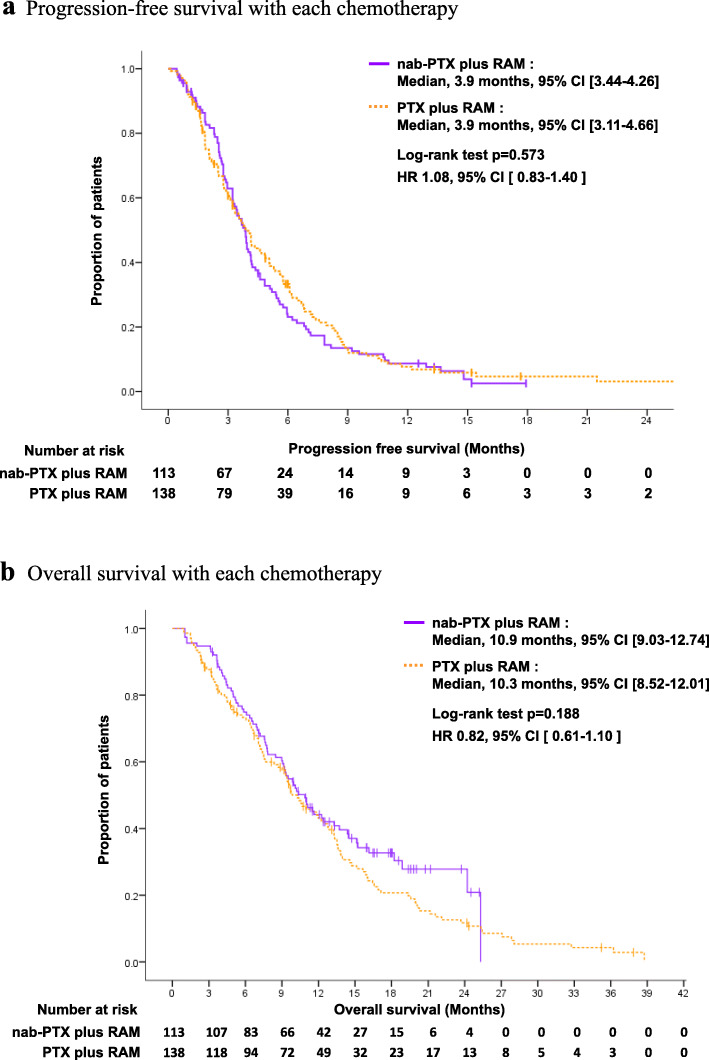
**a** Progression-free survival with each chemotherapy. Solid line. Nab-PTX plus RAM chemotherapy. Dotted line. PTX plus RAM chemotherapy. **b** Overall survival with each chemotherapy. Solid line. Nab-PTX plus RAM chemotherapy. Dotted line. PTX plus RAM chemotherapy

**Fig. 3:**
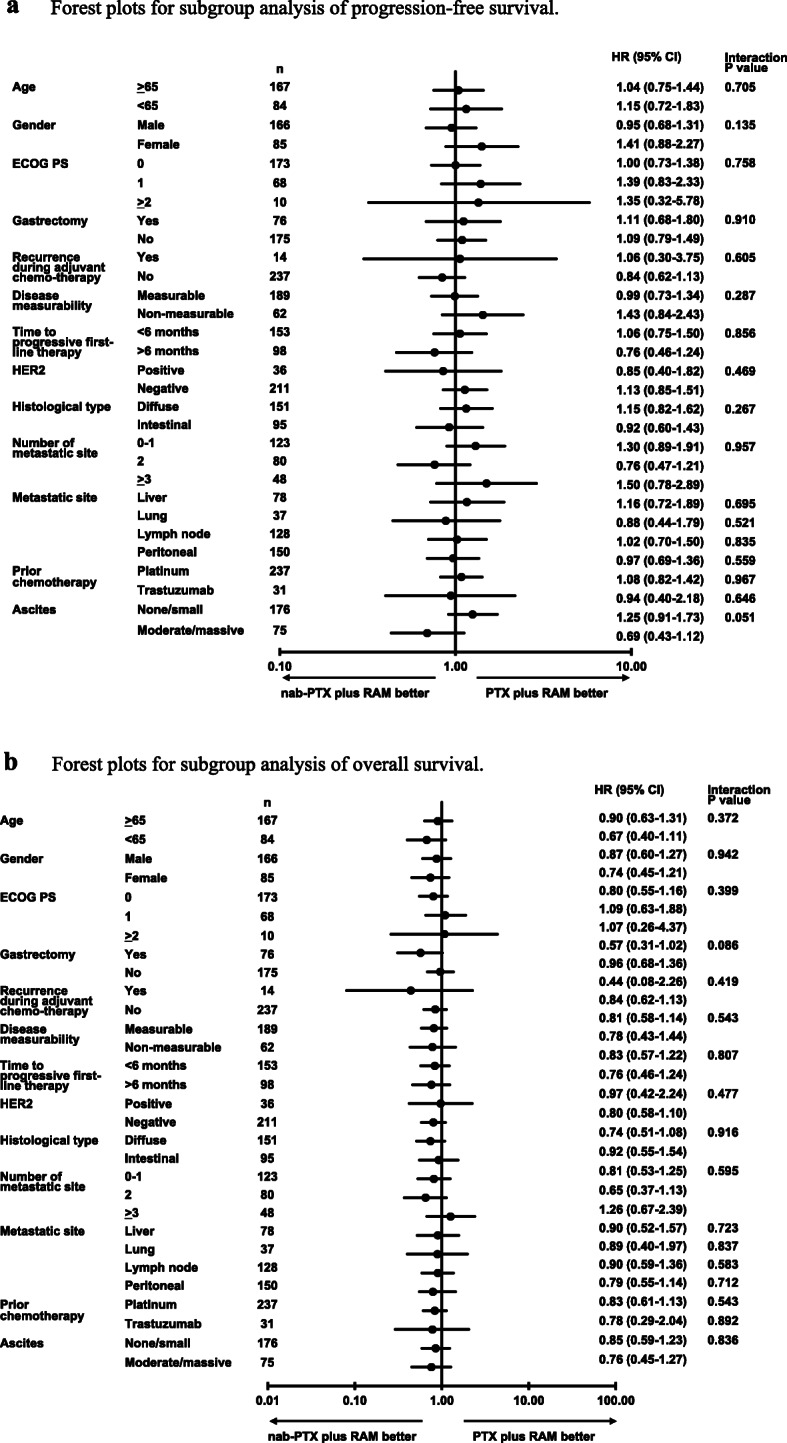
**a** Forest plots for subgroup analyses of progression-free survival. **b** Forest plots for subgroup analyses of overall survival

The median PFS was 3.9 months (95% CI: 3.4–4.3 months) in the nab-PTX plus RAM group and 3.9 months (95% CI: 3.1–4.7 months) in the PTX plus RAM group. PFS was comparable between the two groups (HR: 1.08; 95% CI: 0.83–1.40; *P* = 0.573) (Fig. 2a). The median OS was 10.9 months (95% CI: 9.3–12.7 months) in the nab-PTX plus RAM group and 10.3 months (95% CI: 8.5–12.0 months) in the PTX plus RAM group. There was no significant difference between the two groups (HR 0.82; 95% CI: 0.61–1.10; *P* = 0.188) (Fig. 2b). The results of the subgroup analyses for PFS and OS are shown in Fig. 3. Patients with moderate or massive ascites showed a trend toward favorable outcomes for PFS in the nab-PTX plus RAM group (*P* for interaction = 0.051), whereas there was no obvious trend observed for OS. Most of the other subgroups showed consistent results between PFS and OS.

Eighty-three and 106 patients had measurable lesions in the nab-PTX plus RAM and PTX plus RAM groups, respectively. Among these patients, 28 and 29 patients in the nab-PTX plus RAM and PTX plus RAM groups, respectively, achieved partial response, resulting in a 33.7 and 27.4% ORR, respectively (*P* = 0.385). Of note, higher DCR was observed in the nab-PTX plus RAM group compared with the PTX plus RAM group (81.9% vs. 67.0%, respectively; *P* = 0.016) (Table 3).

### Safety

All patients initially received full-dose RAM. The proportion of patients with initial dose reductions of nab-PTX (54 patients, 47.8%) was significantly higher than that of patients with initial dose reduction of PTX (41 patients, 29.7%) (*P* = 0.003). During treatment, dose reduction or interruption of nab-PTX or PTX occurred in 93 (82.3%) and 98 (71.0%) patients in the nab-PTX plus RAM and PTX plus RAM groups, respectively. The median relative dose intensity (RDI) of nab-PTX in the nab-PTX plus RAM and PTX in the PTX plus RAM group was 57.1 and 61.3%, respectively. There was no difference in RDI of RAM between the nab-PTX plus RAM and PTX plus RAM groups (median: 98.2% vs. 97.6%, respectively).

Treatment-related adverse events (TRAE) are listed in Table 2. Overall, 67.3% (76/113) and 63.8% (88/138) of patients in the nab-PTX plus RAM and PTX plus RAM groups, respectively, experienced grade ≥ 3 TRAEs. The most frequent grade ≥ 3 TRAEs were neutropenia, leukopenia, anemia, thrombocytopenia, hypertension, and febrile neutropenia. Thirty-three (28.0%) and 47 patients (34.1%) in the nab-PTX plus RAM and PTX plus RAM groups, respectively, received granulocyte colony-stimulating factor, without use of granulocyte colony-stimulating factor prophylaxis in either of the groups. Although any grade of anemia, thrombocytopenia, sensory neuropathy, and fatigue were more frequently observed in the nab-PTX plus RAM group, there were no significant differences in grade ≥ 3 TRAEs between the two groups. Hypersensitivity reactions occurred in none of the 113 patients (0%) in the nab-PTX plus RAM group and two of the 138 patients (1.4%) in the PTX plus RAM group. These two patients were emergently hospitalized due to these hypersensitivity reactions.

**Table 2:** Adverse events

	nab-PTX plus RAM		PTX plus RAM		*P* value	
	(*N* = 113)		(*N* = 138)			
	Any grade (%)	>Grade 3 (%)	Any grade (%)	>Grade 3 (%)	Any grade	>Grade 3
All adverse events	112 (99.1)	76 (67.3)	134 (97.1)	88 (63.8)	0.254	0.563
Hematological						
Neutropenia	91 (80.5)	64 (56.6)	108 (78.3)	76 (55.1)	0.659	0.804
Leukopenia	85 (75.2)	34 (30.1)	107 (77.5)	48 (34.8)	0.667	0.43
Anemia	105 (92.9)	8 (7.1)	112 (81.2)	19 (13.8)	0.007	0.089
Thrombocytopenia	43 (38.1)	6 (5.3)	35 (25.4)	4 (2.9)	0.031	0.258
Non-hematological						
Sensory neuropathy	72 (63.7)	2 (1.8)	51 (37.0)	0 (0.0)	0	0.202
Fatigue	42 (37.2)	1 (0.9)	33 (23.9)	0 (0.0)	0.022	0.45
Anorexia	33 (29.2)	1 (0.9)	29 (21.0)	1 (0.7)	0.134	0.699
Nausea	17 (15.0)	2 (1.8)	18 (13.0)	1 (0.7)	0.649	0.425
Diarrhea	7 (6.2)	0 (0.0)	14 (10.1)	0 (0.0)	0.261	–
Stomatitis	10 (8.8)	0 (0.0)	14 (10.1)	1 (0.7)	0.728	0.55
Edema	33 (29.2)	0 (0.0)	32 (23.2)	0 (0.0)	0.279	–
Proteinuria	30 (26.5)	3 (2.7)	29 (21.0)	2 (1.4)	0.304	0.406
Hypertension	30 (26.5)	15 (13.3)	27 (19.6)	9 (6.5)	0.189	0.07
Febrile neutropenia	6 (5.1)	6 (5.1)	13 (9.4)	13 (9.4)	0.221	0.221
Interstitial pneumonia	8 (7.1)	3 (2.7)	5 (3.6)	3 (2.2)	0.219	0.56
Hypersensitivity reaction	0 (0.0)	0 (0.0)	2 (1.4)	2 (1.4)	–	–

*nab-PTX* nanoparticle albumin-bound paclitaxel, *RAM* ramucirumab

**Table 3:** Overall response

Best response	nab-PTX plus RAM		PTX plus RAM		*P* value
	*N* = 83	%	*N* = 106	%	
CR	0	0	0	0	
PR	28	33.7	29	27.4	
SD	40	48.2	42	39.6	
PD	11	13.3	28	26.4	
NE	4	4.8	7	6.6	
ORR	28	33.7	29	27.4	0.385
DCR	68	81.9	71	67	0.016

*nab-PTX* nanoparticle albumin-bound paclitaxel, *RAM* ramucirumab, *CR* complete response, *PR* partial response, *SD* stable disease, *PD* progressive disease, *NE* not evaluated, *ORR* overall response rate, *DCR* disease control rate

TRAEs that led to treatment discontinuation were similar between the two groups: 22.1% (25/113) and 14.5% (20/138) of the patients in the nab-PTX plus RAM and PTX plus RAM groups, respectively. The most common TRAE leading to treatment discontinuation was sensory neuropathy: 3.5% (4/113) and 1.4% (2/138) of the patients in the nab-PTX plus RAM and PTX plus RAM groups, respectively.

## Discussion

To the best of our knowledge, this is the largest cohort study to evaluate the efficacy and safety of nab-PTX plus RAM compared with PTX plus RAM as second-line treatment for patients with AGC. Our study indicated that the combination of nab-PTX plus RAM has a similar efficacy and safety profile to PTX plus RAM in patients with AGC. Although only a single-arm phase II trial has assessed the efficacy and safety of nab-PTX plus RAM, this regimen may be an option for previously treated patients with AGC. This alcohol-free regimen is linked to shorter infusion time and reduced rate of hypersensitivity reactions [13].

There were no significant differences in PFS and ORR between the nab-PTX plus RAM and PTX plus RAM groups. As real-world data, the efficacy observed in the PTX plus RAM group in our study was comparable to that recorded in the RAINBOW study [6]. The PFS and ORR in the nab-PTX plus RAM group were relatively inferior to those reported in the phase II trial of nab-PTX plus RAM for patients with AGC, showing a median PFS of 7.6 months and an ORR of 54.8% [13]. However, a higher proportion of patients who received previous platinum containing regimen and/or < 6 month of duration of first-line chemotherapy, and had peritoneal metastasis were included in our study. The difference in patient characteristics may have led to lower ORR and shorter median PFS compared with those noted in the clinical trial. In terms of OS, there was no significant difference observed between the two groups. However, a higher proportion of patients who received subsequent anti-PD-1/PD-L1 therapy in the nab-PTX plus RAM group contributed to the plateau of the Kaplan–Meier curve at the long-term follow-up compared with that of the PTX plus RAM group. In a subgroup analysis, PFS in patients with moderate/massive ascites tended to be better with nab-PTX plus RAM than PTX plus RAM. The ABSOLUTE trial, which demonstrated the non-inferiority of weekly nab-PTX to weekly PTX for patients with AGC, also suggested an increased efficacy of weekly nab-PTX in patients with ascites or peritoneal metastasis [11, 16]. Although the reason for this remains unclear, higher efficacy of nab-PTX was reported in a gastric cancer preclinical model with subcutaneous and peritoneal xenografts, comparing with PTX [17]. A multicenter randomized phase II P-SELECT trial of nab-PTX plus RAM versus PTX plus RAM as second-line therapy for AGC patients with peritoneal dissemination (WJOG10617G, jRCTs031180022) is underway and may confirm this observation.

The general safety profile of nab-PTX plus RAM was manageable and comparable to that of PTX plus RAM. Although anemia and fatigue (any grade) were more frequent in the nab-PTX plus RAM group, there was no difference in grade ≥ 3 of those adverse events between the two groups, and none of the patients discontinued treatment due to these adverse events. The incidence of sensory neuropathy was also significantly higher in the nab-PTX plus RAM group. However, only 3.5 and 1.4% of patients were forced to discontinue nab-PTX and PTX, respectively. In the phase II trial of nab-PTX plus RAM, 76.7% of patients experienced grade ≥ 3 of neutropenia. In our study, a relatively lower proportion of patients (56.6%) experienced this adverse event. The initial dose reduction of nab-PTX in 47.8% of patients in our study may have resulted in the lower frequency of grade ≥ 3 neutropenia; however, importantly, the median RDI was similar to that observed in the phase II trial [13]. These findings indicated that appropriate dose modification enables treatment continuation, irrespective of nab-PTX plus RAM or PTX plus RAM. Of note, there was no occurrence of hypersensitivity reactions in the nab-PTX plus RAM group with premedication of only chlorpheniramine. Two patients experienced grade 3 hypersensitivity reactions in the PTX plus RAM group and required emergent hospitalization despite adequate premedication. The incidence of infrequent hypersensitivity reactions in patients receiving nab-PTX was consistent with that noted in the ABSOLUTE trial [12]. Nab-PTX was suitable for shorter-time infusion without premedication. The incidence of specific TRAEs related to RAM was similar in both groups, and there were no unexpected TRAEs observed in our study.

This study had several limitations. Firstly, this was a non-randomized retrospective study performed in a single institution with a limited sample size. Secondly, there was a shorter follow-up time in the nab-PTX plus RAM group compared with that of the PTX plus RAM due to the approval of nab-PTX in 2017. Finally, all patients enrolled in this study were Japanese. Although the indication of nab-PTX was approved not for gastric cancer but for breast cancer, non-small cell lung cancer, and adenocarcinoma of pancreas by Food and Drug Administration (FDA) and European Medicines Agency (EMA), recently, a single-arm phase II trial of nab-PTX plus RAM for patients with AGC conducted in the United States of America suggested acceptable safety profiles [18]. These data support the results of our study for the application of this regimen to all patients, regardless of race.

## Conclusion

In conclusion, nab-PTX plus RAM may be a useful treatment option, along with PTX plus RAM, as second-line treatment for patients with AGC, especially in case of known hypersensitivity to PTX or alcohol allergy.

## Supplementary Information

**Additional file 1:** **Table S1.** Subsequent chemotherapy.

## Data Availability

The evaluation data set analyzed in the current study is not available publicly. However, the data are available from the corresponding author on request.

## Abbreviations

Nab-PTX: Nanoparticle albumin-bound paclitaxel
PTX: Paclitaxel
AGC: Advanced gastric cancer
RAM: Ramucirumab
PFS: Progression-free survival
CI: Confidence interval
HR: Hazard ratio
OS: Overall survival
ORR: Overall response rate
DCR: Disease control rate
PD-1: Programmed cell death 1
PD-L1: Programmed cell death ligand 1

## References

[1] Feely J, Soerjomataram I, Ervik M, Dikshit R, Eser S, Mathers C (2014). GLOBOCAN 2012 v1.1, cancer incidence and mortality worldwide: IARS cancer base no. 11.

[2] National Comprehensive Cancer Network (2020). NCCN clinical practice guidelines in oncology gastric cancer. Version 1.

[3] Japanese Gastric Cancer Association (2018). Japanese gastric cancer treatment guideline.

[4] Smyth EC, Verheij M, Allum W, Cunningham D, Cervantes A, Arnold D (2016). Gastric cancer: ESMO clinical practice guidelines for diagnosis, treatment and follow-up. Ann Oncol.

[5] Muro K, Van Cutsem E, Narita Y, Pentheroudakis G, Baba E, Li J (2019). Pan-Asian adapted ESMO clinical practice guidelines for the management of patients with metastatic gastric cancer: a JSMO-ESMO initiative endorsed by CSCO, KSMO, MOS, SSO and TOS. Ann Oncol.

[6] Wilke H, Muro K, Van Cutsem CE, Oh SC, Bodoky G, Shimada Y (2014). Ramucirumab plus paclitaxel versus placebo plus paclitaxel in patients with previously treated advanced gastric cancer or gastro-oesophageal junction adenocarcinoma (RAINBOW): adouble-blind, randomized phase 3 trial. Lancet Oncol.

[7] Shitara K, Muro K, Shimada Y, Hironaka S, Sugimoto N, Komatsu Y (2016). Subgroup aanalysis of the safety and efficacy of ramucirumab in Japanese and Western patients in RAINBOW: a randomised clinical trial in second-line treatment of gastric cancer. Gastric Cancer.

[8] Gelderblom H, Verweij J, Nooter K, Sparreboom A, Cremophor EL (2001). The drawbacks and advantages of vehicle selection for drug formuration. Eur J Cancer.

[9] Weiss RB, Donehower RC, Wiernik PH, Ohnuma T, Gralla RJ, Trump DL (1990). Hypersensitivity reactions from taxol. J Clin Oncol.

[10] Ibrahim NK, Samuels B, Page R, Doval D, Patel KM, Rao SC (2005). Multicenter phase II trial of ABI-007, an albumin-bound paclitaxel, in women with metastatic breast cancer. J Clin Oncol.

[11] Sasaki Y, Nishina T, Yasui H, Goto M, Muro K, Tsuji A (2014). Phase II trial of nanoparticle albumin-bound paclitaxel as second-line chemotherapy for unresectable or recurrent gastric cancer. Cancer Sci.

[12] Shitara K, Takashima A, Fujitani K, Koeda K, Hara H, Nakayama N (2017). Nab-paclitaxel versus solvent-based paclitaxel in patients with previously treated advanced gastric cancer (ABSOLUTE): an open-label, randomized, non-inferiority, phase 3 trial. Lancet Gastroenterol Hepatol.

[13] Bando H, Shimodaira H, Fujitani K, Takashima A, Yamaguchi K, Nakayama N (2018). A phase II study of nab-paclitaxel in combination with ramucirmub in patients with previously treated advanced gastric cancer. Eur J Cancer.

[14] U.S. Department of health and human survices (2010). Common Terminology Criteria for Adverse Events. version 4.03.

[15] Matsumoto H, Kawazoe A, Shimada K, Fukuoka S, Kuboki Y, Bando H (2018). A retrospective study of the safety and efficacy of paclitaxel plus ramucirmab in patients with advanced or recurrent gastric cancer with ascites. BMC Cancer.

[16] Takashima A, Shitara K, Fujitani K, Koeda K, Hara H, Nakayama N (2019). Peritoneal metastasis as a predictive factor for nab-paclitaxel in patients with pretreated advanced gastric cancer: an exploratory analysis of the phase III ABSOLUTE trial. Gastric Cancer.

[17] Kinoshita J, Fushida S, Tsukada T, Oyama K, Watanabe T, Shoji M (2014). Comparative study of the antitumor activity of Nab-paclitaxel and intraperitoneal solvent-based paclitaxel regarding peritoneal metastasis in gastric cancer. Oncol Rep.

[18] Bendell JC, Percent IJ, Weaver RW, Chua CC, Xiong HQ, Cohn AL, et al. phase II study of nab-paclitaxel plus ramucirmab for the second-line treatment of patients with metastatic gastroesophageal cancer. J Clin Oncol. 2020;38:4 suppl 365.

